# Effect of 50% and maximal inspired oxygen concentrations on respiratory variables in isoflurane-anesthetized horses

**DOI:** 10.1186/1746-6148-7-23

**Published:** 2011-06-03

**Authors:** John AE Hubbell, Turi K Aarnes, Richard M Bednarski, Phillip Lerche, William W Muir

**Affiliations:** 1 Deparment of Veterinary Clinical Sciences, College of Veterinary Medicine, The Ohio State University, 601 Vernon L. Tharp St., Columbus, OH 43210, USA; 2 338 W. 7th Ave., Columbus, Ohio 43201, USA

## Abstract

**Background:**

The purpose of this study was to compare the effects of 0.5 fraction of inspired oxygen (FiO_2_) and >0.95 FiO_2 _on pulmonary gas exchange, shunt fraction and oxygen delivery (DO_2_) in dorsally recumbent horses during inhalant anesthesia. The use of 0.5 FiO_2 _has the potential to reduce absorption atelectasis (compared to maximal FiO_2_) and augment alveolar oxygen (O_2_) tensions (compared to ambient air) thereby improving gas exchange and DO_2_. Our hypothesis was that 0.5 FiO_2 _would reduce ventilation-perfusion mismatching and increase the fraction of pulmonary blood flow that is oxygenated, thus improving arterial oxygen content and DO_2_.

**Results:**

Arterial partial pressures of O_2 _were significantly higher than preanesthetic levels at all times during anesthesia in the >0.95 FiO_2 _group. Arterial partial pressures of O_2 _did not change from preanesthetic levels in the 0.5 FiO_2 _group but were significantly lower than in the >0.95 FiO_2 _group from 15 to 90 min of anesthesia. Alveolar to arterial O_2 _tension difference was increased significantly in both groups during anesthesia compared to preanesthetic values. The alveolar to arterial O_2 _tension difference was significantly higher at all times in the >0.95 FiO_2 _group compared to the 0.5 FiO_2 _group. Oxygen delivery did not change from preanesthetic values in either group during anesthesia but was significantly lower than preanesthetic values 10 min after anesthesia in the 0.5 FiO_2 _group. Shunt fraction increased in both groups during anesthesia attaining statistical significance at varying times. Shunt fraction was significantly increased in both groups 10 min after anesthesia but was not different between groups. Alveolar dead space ventilation increased after 3 hr of anesthesia in both groups.

**Conclusions:**

Reducing FiO_2 _did not change alveolar dead space ventilation or shunt fraction in dorsally recumbent, mechanically ventilated horses during 3 hr of isoflurane anesthesia. Reducing FiO_2 _in dorsally recumbent isoflurane anesthetized horses does not improve oxygenation or oxygen delivery.

## Background

Maximal FiO_2 _values (>95%) have been administered to horses to maintain or improve the arterial partial pressure of oxygen (PaO_2_) and tissue oxygenation since the advent of inhalant anesthesia [1]. Maximal FiO_2 _concentrations are used in the belief that their use optimizes arterial O_2 _content (CaO_2_) and O_2 _delivery (DO_2_) to tissues providing cardiac output (Q) is maintained [2]. Lower than expected (subnormal) PaO_2 _values are frequently encountered during equine inhalant anesthesia despite the use of maximal FiO_2 _[3-6]. Subnormal PaO_2 _values usually develop during the first 30 to 90 min of anesthesia, continue for the duration of anesthesia, and resolve within 60 min of recovery to standing [4]. The 5 mechanisms producing subnormal PaO_2 _values are decreased FiO_2_, diffusion impairment, vascular shunting (true shunt), hypoventilation, and ventilation-perfusion mismatching (pseudo shunt) [4]. Decreased FiO_2_, diffusion impairment, and vascular shunts are unlikely to contribute to subnormal PaO_2 _during inhalation anesthesia in normal horses. Hypoventilation [arterial partial pressures of carbon dioxide (PaCO_2_) >5.92 kPa (45 mm Hg)] is a common occurrence during equine anesthesia because virtually all anesthetic drugs cause respiratory depression. Hypoventilation is easily corrected by mechanical ventilation [7]. The use of mechanical ventilators, mechanical ventilation and increased FiO_2 _probably reduces the incidence of subnormal PaO_2 _values however a significant number of horses continue to experience subnormal PaO_2 _despite normocapnia and increased FiO_2 _[2,5,6].

The major cause of subnormal PaO_2 _values in anesthetized horses is ventilation-perfusion mismatching within the lung parenchyma [8]. Functional residual capacity and residual volume are reduced in recumbent horses during inhalant anesthesia [3]. The distribution of ventilation is shifted to non-dependent areas of lung and altered by changes in diaphragmatic function and mechanical ventilation [9-13]. The shifting of the distribution of lung ventilation uncouples ventilation and perfusion because lung perfusion remains primarily caudal-dorsal regardless of body posture [14-16]. Ventilation-perfusion mismatching is exacerbated by atelectasis because collapsed alveoli continue to be perfused with little or no gas exchange. The two primary causes of atelectasis are compression (from overlying abdominal contents) and absorption (collapse due to absorption of the alveolar gas). Compression atelectasis is more pronounced in dorsally recumbent horses compared to other species due to its long, sloping diaphragm [17]. The development of compression atelectasis can be partially offset or delayed by initiating mechanical ventilation early during the anesthetic period but predicted PaO_2 _values are not always achieved [2]. Ventilatory recruitment maneuvers including the use of positive end expiratory pressure (PEEP) have been investigated in horses, however improvements are inconsistent and only occur when PEEP values that compromise Q are employed [12,18,19].

The relationship between PaO_2 _and hemoglobin saturation varies with blood temperature, pH and intracellular factors [13,20]. Although equine hemoglobin is more than 90% saturated at PaO_2 _values greater than 9.2 kPa (70 mm Hg), there is rapid desaturation at values below 7.9 kPa (60 mm Hg) [13]. One author has provided data from anesthetized horses demonstrating that DO_2 _decreases when PaO_2 _decreases, and that PaO_2 _values less than 5.6 kPa (50 mm Hg) are associated with decreases in venous partial pressure of oxygen (PvO_2_), an indicator of the adequacy of DO_2 _[21].

The recognition that atelectasis contributes to ventilation-perfusion mismatching, intrapulmonary shunt and subnormal PaO_2 _values has raised questions about the use of maximal FiO_2 _[22,23]. Absorption atelectasis is exacerbated by administering maximal FiO_2 _because O_2 _is more readily absorbed than other, less soluble, gases such as nitrogen. Further, studies in humans suggest that the use of maximal FiO_2 _promotes alveolar damage, reduces cardiac index, and increases peripheral vascular resistance [24,25]. The use of 0.5 FiO_2 _could reduce absorption atelectasis and augment alveolar O_2 _tensions (compared to ambient air) thereby improving gas exchange and DO_2_. The purpose of this study was to compare the effects of 0.5 FiO_2 _and >0.95% FiO_2 _on pulmonary gas exchange, shunt fraction (Qs/Qt) and DO_2 _in dorsally recumbent horses during inhalant anesthesia. Our hypothesis was that 0.5 FiO_2 _would reduce ventilation-perfusion mismatching and increase the fraction of pulmonary blood flow that is oxygenated, thus improving CaO_2 _and DO_2_.

## Results

All horses completed all phases of the experiment. Initial flow rates for the 0.5 FiO_2 _group were 3 L/min of O_2 _and 6 L/min of air. Flow rates required to maintain 0.5 FiO_2 _when the total flow rate was set at 9 mL/kg bwt/min ranged from 1.8 to 3 L/min for O_2 _and 3 to 4 L/min for air. All horses were administered dobutamine during each trial (dose range 0.5-1 μg/kg/min). One horse developed atrial fibrillation 30 min after induction of anesthesia during the second procedure (>0.95 FiO_2_). Atrial fibrillation continued throughout anesthesia and an electrocardiogram confirmed normal sinus rhythm 1 hr after the horse stood. Blood pressures and heart rates in this horse were similar to other horses in this treatment group and ranged from 30 to 36 bpm prior to the onset of atrial fibrillation and from 35 to 56 bpm after the onset of atrial fibrillation.

There were no changes or differences in right atrial pressure (RAP), mean pulmonary artery pressure (MPAP), cardiac output (Q), cardiac index (CI), or systemic vascular resistance (SVR) over time or between groups (Table 1). Dobutamine-augmented arterial blood pressures were significantly decreased from preanesthetic values in both groups from 15 to 180 min after induction to anesthesia. Arterial blood pressure was significantly higher than preanesthetic values 30 min after standing in the 0.5 FiO_2 _group but only mean arterial blood pressure (MABP) was significantly higher in the >0.95 FiO_2 _group at this interval. There were no differences between groups. Respiratory rate was controlled at a rate of 4 - 5 breaths/min during anesthesia. Respiratory rate was significantly decreased compared to preanesthetic levels 10 min after anesthesia in both groups.

**Table 1:** Hemodynamic and patient variables from isoflurane-anesthetized horses (n = 5) breathing 50% or maximal oxygen concentrations

Parameter	FiO2	-5 min	15 min	30 min	60 min	90 min	120 min	150 min	180 min	+10 min	Stand + 30 min
HR (beats/min)	50%	35 ± 2	32 ± 1	39 ± 2	40 ± 3	46 ± 3	43 ± 2	39 ± 3	39 ± 3	38 ± 4	38 ± 2
	>95%	42 ± 6	35 ± 3	37 ± 3	39 ± 5	39 ± 3	42 ± 3	41 ± 4	43 ± 4	34 ± 4	45 ± 5
RR (breaths/min)	50%	26 ± 3	6 ± 0^a^	5 ± 0^a^	5 ± 0^a^	4 ± 0^a^	4 ± 0^a^	4 ± 0^a^	4 ± 0^a^	12 ± 1^a^	20 ± 5
	>95%	27 ± 5	6 ± 0^a^	5 ± 0^a^	5 ± 0^a^	5 ± 0^a^	5 ± 0^a^	5 ± 0^a^	5 ± 0^a^	12 ± 4^a^	17 ± 2
PIP (cmH_2_0)	50%	-----	29 ± 4	30 ± 3	31 ± 3	31 ± 3	32 ± 3	32 ± 3	33 ± 3	-----	-----
	>95%	-----	30 ± 4	29 ± 3	31 ± 2	32 ± 3	31 ± 3	32 ± 3	31 ± 3	-----	-----
EtCO2 (kPa)	50%	-----	4.18 ± 0.21	4.08 ± 0.13	4.18 ± 0.13	4.42 ± 0.18	4.21 ± 0.04	4.05 ± 0.10	3.97 ± 0.11	-----	-----
	>95%	-----	3.92 ± 0.33	4.32 ± 0.30	4.47 ± 0. 62	4.34 ± 0.12	4.21 ± 0.09	4.03 ± 0.12	3.82 ± 0.12	-----	-----
SABP (mmHg)	50%	143 ± 9	109 ± 6^a^	88 ± 5^a^	107 ± 4^a^	106 ± 3^a^	105 ± 2^a^	100 ± 1^a^	104 ± 2^a^	128 ± 5	176 ± 8^a^
	>95%	147 ± 7	106 ± 8^a^	95 ± 4^a^	103 ± 6^a^	103 ± 7^a^	104 ± 6^a^	105 ± 4^a^	102 ± 5^a^	129 ± 7	172 ± 10
MABP (mmHg)	50%	110 ± 3	82 ± 5^a^	66 ± 4^a^	80 ± 2^a^	85 ± 1^a^	84 ± 2^a^	84 ± 3^a^	85 ± 1^a^	109 ± 4	138 ± 7^a^
	>95%	112 ± 6	82 ± 9^a^	69 ± 3^a^	79 ± 5^a^	80 ± 4^a^	81 ± 4^a^	84 ± 4^a^	82 ± 2^a^	106 ± 4	136 ± 6^a^
DABP (mmHg)	50%	87 ± 5	69 ± 4	54 ± 3^a^	68 ± 2	72 ± 3	73 ± 3	71 ± 4	76 ± 2	96 ± 3	123 ± 11^a^
	>95%	93 ± 6	69 ± 8	58 ± 4^a^	66 ± 4^a^	67 ± 3^a^	72 ± 4	73 ± 4	70 ± 3	97 ± 4	115 ± 6
MRAP (mmHg)	50%	14 ± 3	6 ± 2	9 ± 1	10 ± 2	9 ± 2	12 ± 1	12 ± 1	13 ± 2	24 ± 1	15 ± 3
	>95%	13 ± 2	10 ± 2	13 ± 1	12 ± 2	11 ± 2	12 ± 3	14 ± 3	14 ± 3	21 ± 4	14 ± 3
MPAP (mmHg)	50%	30 ± 4	14 ± 1	17 ± 0	20 ± 2	23 ± 2	24 ± 3	24 ± 2	23 ± 2	36 ± 2	32 ± 3
	>95%	31 ± 2	21 ± 5	23 ± 4	26 ± 5	26 ± 5	26 ± 5	26 ± 5	26 ± 5	34 ± 5	34 ± 4
Q (L/min)	50%	34.2 ± 5.6	25.4 ± 2.8	28.1 ± 3.4	31.5 ± 2.8	32.9 ± 3.8	25.2 ± 3.3	23.3 ± 2.4	22.7 ± 1.5	19.9 ± 1.4	36.5 ± 3.6
	>95%	34.2 ± 3.6	22.5 ± 1.1	24.2 ± 1.5	30.1 ± 1.9	26.1 ± 3.6	25.2 ± 3.1	21.1 ± 0.9	21.3 ± 0.9	21.8 ± 1.5	40.2 ± 3.9
CI (L/kg bwt/min)	50%	0.056 ± 0.010	0.041 ± 0.004	0.045 ± 0.005	0.051 ± 0.005	0.053 ± 0.005	0.041 ± 0.005	0.038 ± 0.004	0.037 ± 0.0002	0.032 ± 0.002	0.059 ± 0.006
	>95%	0.056 ± 0.005	0.037 ± 0.001	0.040 ± 0.003	0.049 ± 0.003	0.043 ± 0.006	0.041 ± 0.005	0.035 ± 0.001	0.035 ± 0.001	0.036 ± 0.002	0.066 ± 0.007
SVR (dyn sec cm^-5^)	50%	248 ± 33	249 ± 34	172 ± 23	182 ± 13	195 ± 21	245 ± 33	258 ± 26	261 ± 22	347 ± 17	282 ± 36
	>95%	234 ± 13	256 ± 26	190 ± 25	182 ± 15	227 ± 35	227 ± 21	268 ± 14	256 ± 13	318 ± 24	251 ± 22

Data are presented as mean ± standard error of the mean. HR, heart rate; RR, respiratory rate; PIP, peak inspiratory pressure; EtCO_2_, end-tidal CO_2_; SABP, systolic arterial blood pressure; MABP, mean arterial blood pressure; DABP, diastolic arterial blood pressure; MRAP, mean right atrial pressure; MPAP, mean pulmonary artery pressure; Q, cardiac output; CI, cardiac index; SVR, systemic vascular resistance. Time points: -5, 15, 30, 60, 90, 120, 150, and 180 represent min prior to and after induction of anesthesia; +10 min represents 10 min after disconnection from the anesthesia machine; and Stand +30 min represents 30 min after the horses stood in recovery.

a Within a treatment, value is significantly different than the -5 min value for this variable.

Arterial partial pressures of O_2 _were significantly higher than preanesthetic values at all times during anesthesia in the >0.95 FiO_2 _group (Table 2). Arterial partial pressures of O_2 _did not change from preanesthetic values in the 0.5 FiO_2 _group and were significantly lower than the >0.95 FiO_2 _group at the 15, 30, 60, and 90 min measurement intervals (Table 2). Arterial hemoglobin saturations were not different from preanesthetic levels or between groups at any point during anesthesia in either group but were significantly lower than preanesthetic levels at the 10 min postanesthetic measurement interval in both groups. Venous partial pressure of oxygen was increased compared to baseline from 60 to 120 min in the >0.95 FiO_2 _group but there were no differences between groups. Venous lactate concentrations were increased compared to preanesthetic concentrations from 90 to 180 min during anesthesia and 10 min after anesthesia in the 0.5 FiO_2 _group and at 60, 150, and 180 min during anesthesia and 10 min after anesthesia in >0.95 FiO_2 _group. There were no differences in venous lactate concentrations between groups (Table 2).

**Table 2:** Blood variables from isoflurane-anesthetized horses (n = 5) breathing 50% or maximal oxygen concentrations

Parameter	FiO2	-5 min	15 min	30 min	60 min	90 min	120 min	150 min	180 min	+10 min	Stand + 30 min
pHa	50%	7.446 ± 0.016	7.448 ± 0.014	7.461 ± 0.011	7.429 ± 0.008	7.409 ± 0.014	7.414 ± 0.016	7.424 ± 0.012	7.412 ± 0.016	7.375 ± 0.022	7.425 ± 0.011
	>95%	7.428 ± 0.013	7.461 ± 0.031	7.437 ± 0.032	7.424 ± 0.027	7.431 ± 0.021	7.424 ± 0.018	7.432 ± 0.014	7.441 ± 0.017	7.386 ± 0.031	7.438 ± 0.005
PaO_2 _(kPa)	50%	12.06 ± 0.92	12.15 ± 1.24^b^	12.55 ± 0.98^b^	13.73 ± 01.16^b^	13.48 ± 1.39^b^	12.55 ± 1.54	10.89 ± 1.43	10.29 ± 1.48	5.60 ± 0.24	12.00 ± 0.35
	>95%	12.07 ± 0.53	36.03 ± 4.02^a,b^	32.13 ± 4.78^a,b^	36.47 ± 5.93^a,b^	34.18 ± 5.24^a,b^	31.37 ± 6.32^a^	29.20 ± 6.12	28.61 ± 6.34	5.92 ± 0.37	10.39 ± 1.07
Arterial Hb (g/dL)	50%	13.2 ± 1.7	12.4 ± 0.4	12.0 ± 0.5	14.1 ± 0.2	13.8 ± 0.2	13.5 ± 0.5	13.0 ± 0.7	12.8 ± 0.5	12.6 ± 0.3	11.8 ± 0.5
	>95%	12.0 ± 0.5	11.9 ± 0.4	13.0 ± 0.6	13.7 ± 0.8	13.7 ± 0.3	13.6 ± 0.4	13.7 ± 0.7	13.3 ± 0.7	12.2 ± 0.6	11.5 ± 0.3
PaCO_2 _(kPa)	50%	4.84 ± 0.38	4.79 ± 0.30	4.63 ± 0.20	5.08 ± 0.08	5.53 ± 0.36	5.26 ± 0.25	5.11 ± 0.10	5.52 ± 0.32	6.76 ± 0.53^a^	5.59 ± 0.23
	>95%	5.36 ± 0.23	4.57 ± 0.45	5.25 ± 0.37	5.37 ± 0.38	5.39 ± 0.31	5.51 ± 0.16	5.57 ± 0.18	5.42 ± 0.26	6.70 ± 0.76	5.76 ± 0.20
PaHCO_3 _(mmol/L)	50%	26 ± 1	26 ± 0	26 ± 1	26 ± 0	26 ± 1	25 ± 1	25 ± 1	26 ± 1	27 ± 1	27 ± 1
	>95%	27 ± 1	25 ± 1	27 ± 1	26 ± 1	27 ± 0	27 ± 1	28 ± 1	28 ± 1	28 ± 0	29 ± 1
SaO_2 _(%)	50%	96.3 ± 0.5	95.8 ± 0.8	96.4 ± 0.4	96.4 ± 0.5	96.2 ± 0.4	95.6 ± 0.9	94.7 ± 1.3	93.4 ± 1.8	81.5 ± 1.7^a^	96.5 ± 0.2
	>95%	96.8 ± 0.3	97.8 ± 0.1	97.6 ± 0.1	97.6 ± 0.2	97.6 ± 0.1	97.5 ± 0.2	97.4 ± 0.3	97.0 ± 0.7	84.0 ± 2.2^a^	95.1 ± 0.9
pHv	50%	7.402 ± 0.010	7.418 ± 0.011	7.433 ± 0.008	7.408 ± 0.008	7.400 ± 0.011	7.398 ± 0.011	7.397 ± 0.013	7.392 ± 0.012	7.347 ± 0.015	7.387 ± 0.008
	>95%	7.399 ± 0.006	7.422 ± 0.027	7.408 ± 0.027	7.401 ± 0.024	7.397 ± 0.020	7.398 ± 0.016	7.402 ± 0.015	7.407 ± 0.016	7.352 ± 0.019	7.397 ± 0.004
PvO_2 _(kPa)	50%	4.44 ± 0.41	4.44 ± 0.09	4.61 ± 0.12	5.55 ± 0.22	5.64 ± 0.14	5.34 ± 0.27	4.95 ± 0.33	4.85 ± 0.29	3.72 ± 0.18	3.74 ± 0.21
	>95%	4.11 ± 0.10	4.96 ± 0.11	5.08 ± 0.16	5.88 ± 0.34^a^	5.79 ± 0.43^a^	5.71 ± 0.48^a^	5.43 ± 0.35	5.32 ± 0.34	3.73 ± 0.19	3.59 ± 0.26
VLactate (mmol/L)	50%	0.5 ± 0	1.1 ± 0.1	1.2 ± 0.1	1.3 ± 0.1	1.4 ± 0.1^a^	1.4 ± 0.1^a^	1.5 ± 0.1^a^	1.5 ± 0.1^a^	1.4 ± 0.1^a^	1.1 ± 0.2
	>95%	0.5 ± 0.1	1.2 ± 0.1	1.4 ± 0.1	1.4 ± 0.1^a^	1.3 ± 0.1	1.4 ± 0.1	1.5 ± 0.1^a^	1.5 ± 0.1^a^	1.4 ± 0.1^a^	1.2 ± 0.5

Data are presented as mean ± standard error of the mean. pHa, arterial pH; PaO_2_, arterial partial pressure of oxygen; Arterial Hb, arterial hemoglobin; PaCO_2_, arterial partial pressure of carbon dioxide; PaHCO_3_, arterial bicarbonate; SaO_2_, arterial oxygen saturation; pHv, venous pH; PvO_2_, venous partial pressure of oxygen; vLactate, venous lactate. Time points: -5, 15, 30, 60, 90, 120, 150, and 180 represent min prior to and after induction of anesthesia; +10 min represents 10 min after disconnection from the anesthesia machine; and Stand +30 min represents 30 min after the horses stood in recovery.

a Within a treatment, value is significantly different than the -5 min value for this variable.

b Within this time point, value is significantly different than other treatment

Arterial O_2 _content (CaO_2_), venous O_2 _content (CvO_2_), and oxygen extraction ratio (O_2_ER) did not change during the experiment (Table 3). Oxygen consumption (VO_2_) decreased after induction to anesthesia, becoming significant from 60 to 180 min in the 0.5 FiO_2 _group and at 15 min in the >0.95 FiO_2 _group. Oxygen consumption was not different between groups. Alveolar to arterial O_2 _tension differences (P_(A-a)_O_2_) were significantly increased during anesthesia compared to preanesthetic values in both groups and tension differences in the >0.95 FiO_2 _group were significantly higher than in 0.5 FiO_2 _group at all measurement intervals. Oxygen delivery did not change from preanesthetic levels during anesthesia in either group but was significantly lower than preanesthetic levels 10 min after anesthesia in the 0.5 FiO_2 _group. Shunt fraction increased in both groups during anesthesia and was significant at 60, 90, 150, and 180 min in the 0.5 FiO_2 _group and from 15 to 150 min in the >0.95 FiO_2 _group. Shunt fraction was significantly increased in both groups 10 min after anesthesia. Shunt fraction was not different between groups at any time point. Alveolar dead space ventilation (V_d_/V_t_) was increased after 3 hr of anesthesia in both groups compared to 15 min after induction.

**Table 3:** Oxygen variables from isoflurane-anesthetized horses (n = 5) breathing 50% or maximal oxygen concentrations

Parameter	FiO2	-5 min	15 min	30 min	60 min	90 min	120 min	150 min	180 min	+10 min	Stand + 30 min
CaO_2 _(mL/dL)	50%	17.9 ± 2.1	16.7 ± 0.5	16.4 ± 0.7	19.3 ± 0.3	18.7 ± 0.3	18.3 ± 0.7	17.4 ± 1.1	16.9 ± 1.0	14.4 ± 0.4	16.2 ± 0.6
	>95%	16.4 ± 0.7	17.0 ± 0.5	18.3 ± 1.0	19.4 ± 1.2	19.4 ± 0.6	19.1 ± 0.7	19.2 ± 1.1	18.6 ± 1.2	14.3 ± 0.8	15.4 ± 0.6
CvO_2 _(mL/dL)	50%	13.0 ± 1.7	13.1 ± 0.6	13.2 ± 0.5	16.9 ± 0.6	16.6 ± 0.3	15.5 ± 0.9	14.9 ± 1.5	14.1 ± 1.3	9.9 ± 0.7	10.2 ± 0.7
	>95%	11.3 ± 0.5	14.3 ± 0.5	15.1 ± 1.0	16.5 ± 1.4	16.4 ± 1.2	15.9 ± 1.3	15.4 ± 1.3	14.7 ± 1.3	9.7 ± 0.8	9.3 ± 1.2
O_2_ER (%)	50%	27 ± 4	22 ± 2	19 ± 3	12 ± 3	11 ± 1	15 ± 3	15 ± 4	17 ± 3	32 ± 3	37 ± 4
	>95%	31 ± 1	16 ± 2	18 ± 4	15 ± 3	16 ± 4	17 ± 5	20 ± 3	21 ± 3	32 ± 3	40 ± 6
VO_2 _(mL/kg bwt/min)	50%	2.9 ± 1	1.5 ± 0.2	1.4 ± 0.2	1.1 ± 0.2^a^	1.1 ± 0.1^a^	1.0 ± 0.1^a^	0.9 ± 0.1^a^	1.0 ± 0.1^a^	1.4 ± 0.1	3.5 ± 0.5
	>95%	2.8 ± 0.2	1.0 ± 0.1^a^	1.3 ± 0.2	1.4 ± 0.2	1.1 ± 0.2	1.2 ± 0.2	1.3 ± 0.1	1.3 ± 0.1	1.6 ± 0.1	4.0 ± 0.8
C(a-v)O_2 _(mL/dL)	50%	4.87 ± 0.79	3.61 ± 0.40	3.20 ± 0.59	2.34 ± 0.49	2.12 ± 0.29	2.77 ± 0.48	2.53 ± 0.56	2.73 ± 0.38	4.52 ± 0.28	5.93 ± 0.65
	>95%	5.12 ± 0.32	2.75 ± 0.40	3.28 ± 0.65	2.86 ± 0.49	3.00 ± 0.74	3.18 ± 0.84	3.75 ± 0.52	3.83 ± 0.46	4.61 ± 0.51	6.08 ± 0.80
PaO_2_-IO_2 _ratio	50%	0.64 ± 0.05	0.27 ± 0.03^a^	0.28 ± 0.02^a^	0.30 ± 0.02^a^	0.29 ± 0.03^a^	0.27 ± 0.03^a^	0.23 ± 0.03^a^	0.22 ± 0.03^a^	0.30 ± 0.01^a^	0.64 ± 0.02
	>95%	0.64 ± 0.03	0.41 ± 0.05	0.36 ± 0.05^a^	0.41 ± 0.07	0.38 ± 0.06^a^	0.35 ± 0.07^a^	0.32 ± 0.07^a^	0.32 ± 0.07^a^	0.32 ± 0.02^a^	0.55 ± 0.06
P(A-a)O_2 _(kPa)	50%	1.84 ± 1.09	27.51 ± 2.07^a,b^	27.12 ± 1.00^a,b^	26.52 ± 1.05^a,b^	26.79 ± 1.29^a,b^	27.85 ± 1.23^a,b^	30.26 ± 1.60^a,b^	29.81 ± 1.44^a,b^	5.99 ± 0.67	0.99 ± 0.20
	>95%	1.20 ± 0.31	45.93 ± 3.90^a,b^	51.07 ± 4.51^a,b^	46.77 ± 5.97^a,b^	49.22 ± 5.26^a,b^	52.08 ± 6.38^a,b^	54.36 ± 6.31^a,b^	55.33 ± 6.48^a,b^	5.74 ± 0.90	2.40 ± 1.04
DO_2 _(mL/kg bwt/min)	50%	10.82 ± 3.59	6.84 ± 0.60	7.44 ± 0.84	9.82 ± 0.85	9.91 ± 0.94	7.48 ± 1.07	6.71 ± 1.00	6.24 ± 0.61	4.62 ± 0.26^a^	9.47 ± 0.78
	>95%	9.16 ± 1.00	6.26 ± 0.24	7.24 ± 0.49	9.55 ± 0.89	8.34 ± 1.19	7.91 ± 1.03	6.65 ± 0.50	6.47 ± 0.44	5.11 ± 0.41	10.20 ± 1.19
Qs/Qt (%)	50%	13 ± 2	28 ± 4	30 ± 5	39 ± 8^a^	40 ± 6^a^	36 ± 4	40 ± 4^a^	40 ± 3^a^	43 ± 4^a^	9 ± 1
	>95%	9 ± 1	35 ± 4^a^	36 ± 6^a^	36 ± 4^a^	38 ± 5^a^	37 ± 4^a^	33 ± 3^a^	32 ± 3	38 ± 5^a^	12 ± 2
Vd/Vt (%)	50%	-----	11.9 ± 4.4	11.7 ± 1.3	17.6 ± 1.5	19.4 ± 2.6	19.4 ± 3.1	20.5 ± 2.4	27.2 ± 3.9^c^	-----	-----
	>95%	-----	13.6 ± 3.2	17.7 ± 1.9	16.4 ± 2.2	18.7 ± 3.8	23.4 ± 2.3	27.6 ± 2.3	29.2 ± 2.2^c^	-----	-----

Data are presented as mean ± standard error of the mean. CaO_2_, arterial oxygen content; CvO_2_, venous oxygen content; O_2_ER, oxygen extraction ratio; VO_2_, oxygen consumption; C(a-v)O_2_, oxygen content difference; PaO_2_-IO_2 _ratio, arterial oxygen tension to inspired oxygen tension ratio; P(A-a)O_2_, alveolar arterial oxygen tension difference; DO_2_, oxygen delivery; Qs/Qt, shunt fraction; Vd/Vt, alveolar dead space. Time points: -5, 15, 30, 60, 90, 120, 150, and 180 represent min prior to and after induction of anesthesia; +10 min represents 10 min after disconnection from the anesthesia machine; and Stand +30 min represents 30 min after the horses stood in recovery.

a Within a treatment, value is significantly different than the -5 min value for this variable.

b Within this time point, value is significantly different than other treatment

c With a treatment, value is significantly different from the 15 min value for this variable

Horses stood at 96+/-13 min and 91+/-10 min in the >0.95 FiO_2 _and 0.5 FiO_2 _groups, respectively (Table 4). Times to first movement, extubation, first attempt to sternal recumbency, sternal recumbency, first attempt to stand, and standing were not different between groups (Table 4). Median number of attempts to stand was 1 and median recovery score was 3 in both groups.

**Table 4:** Recovery variables of isoflurane-anesthetized horses (n = 5) breathing 50% or maximal oxygen concentrations

Parameter	FiO2 > 95%	FiO2 50%
Time to first movement (min)^a^	48 ± 7	57 ± 5
Time to extubation (min)^a^	31 ± 7	34 ± 6
Time to first attempt to sternal recumbency (min)^a^	73 ± 17	67 ± 6
Time to sternal recumbency (min)^a^	88 ± 13	76 ± 9
Time to first attempt to stand (min)^a^	94 ± 14	84 ± 9
Time to standing (min)^a^	96 ± 13	91 ± 10
Number of attempts to stand^b^	1 (1 - 3)	1 (1 - 5)
Median Recovery Score^b^	3 (1 - 4)	3 (2 - 7)

a Data are presented as mean ± standard error of the mean

b Data are presented as median (range)

## Discussion

The use of 0.5 FiO_2 _in dorsally-recumbent isoflurane-anesthetized horses did not reduce Qs/Qt or Vd/Vt compared to >0.95 FiO_2_. Arterial partial pressures of O_2 _and P_(A-a)_O_2 _were higher in horses breathing >0.95 FiO_2 _but DO_2 _was not different between groups during 3 hr of anesthesia or after anesthesia. Reduction of FiO_2 _from >95% to 50% did not improve pulmonary gas exchange or oxygenation.

The results of this study support the hypothesis that the development of Qs/Qt mismatching from compression atelectasis is the most likely cause of impaired arterial oxygenation during inhalant anesthesia in the horse [8]. Shunt fraction, P_(A-a)_O_2_, and Vd/Vt increased in both groups after induction to anesthesia in our study. Our results differ from two previous reports that found a greater Qs/Qt in horses that breathed >0.95 FiO_2 _compared to horses that breathed air during intravenous anesthesia [8] and an increase in Vd/Vt when >0.85 FiO_2 _was compared to 0.35 FiO_2 _in spontaneously breathing, halothane-anesthetized horses [26]. In agreement with our results, the administration of varying helium-oxygen mixtures did not change Qs/Qt but resulted in progressive increases in P_(A-a)_O_2 _and PaO_2 _as FiO_2 _increased [27]. Differences between our results and those of others may be due to the FiO_2 _selected (0.5 versus 0.2 to 0.35), the effects of intravenous and different inhalant anesthetics (isoflurane versus no inhalant or halothane), the duration of anesthesia, the mode of ventilation (controlled ventilation versus spontaneous ventilation), or body position (dorsal recumbency versus lateral recumbency). Positive pressure ventilation and inhalant anesthetics alter the distribution of ventilation in the anesthetized horse but most have accepted this limitation of the two techniques in the interest of normalization of PaCO_2 _and the maintenance of consistent anesthetic depth [9-12]. Body position is a primary determinant of oxygenation in anesthetized horses with dorsal recumbency consistently associated with larger decrements in pulmonary function and gas exchange [2,9,28,29]. The cause for the decrements in pulmonary function has classically been attributed to a gravity-dependent shift in blood flow and a shift in ventilation to less dependent lung zones [30]. This description has been challenged by several studies that have demonstrated that only small shifts in blood flow occur in anesthetized horses after changes of body position [15,29,31]. We purposefully selected overweight horses and placed them in dorsal recumbency for an extended period in order to maximize the potential for changes in FiO_2 _to improve gas exchange and reduce hypoxemia [5,32-34].

Similar to others we confirmed that >0.95 FiO_2 _is associated with greater PaO_2 _and P_(A-a)_O_2 _values than the those produced by 0.2 to 0.5 FiO_2 _and that CaO_2 _and DO_2 _may not be markedly different if arterial hemoglobin remains fully saturated [23,26,27]. Step-wise increases in FiO_2 _to titrate PaO_2 _and CaO_2 _have been proposed [27] but such an approach requires multiple gas sources, the ability to determine FiO_2 _and frequent sampling of arterial blood for arterial blood gas analysis. The differences in PaO_2 _and P_(A-a)_O_2 _between the two groups in the face of no difference in calculated Qs/Qt is a function of the sigmoid shape of the oxyhemoglobin saturation curve and highlights the importance of considering both PaO_2 _and CaO_2 _when assessing the significance of changes in the ranges of PaO_2 _and Qs/Qt routinely encountered in the anesthetized horse [35].

The incidence of subnormal PaO_2 _values and hypoxemia in dorsally recumbent anesthetized horses is a recurring problem during equine anesthesia and has been associated with body shape, body weight, low pulse pressures, emergency case status, and male gender [5,6,33,34]. It has been suggested that the application of controlled ventilation immediately after induction to anesthesia is more likely to maintain PaO_2 _during anesthesia but controlled ventilation may decrease Q and thus DO_2 _[2,21]. Additional proposed methods of improving oxygenation in anesthetized horses include PEEP, inhalation of aerosolized bronchodilators such as albuterol, selective mechanical ventilation of dependent lung regions and breathing helium-oxygen mixtures [18,27,36,37]. Positive end expiratory pressures of at least 10 cm of water may improve CaO_2 _without changing Qs/Qt but Q is reduced unless fluids and cardiac inotropes (ex. dobutamine) are administered [17,18]. Aerosolized albuterol is reported to increase PaO_2 _but its effects are inconsistent in the authors' and other's experience regarding efficacy and consistency of effect [36,38]. Selective mechanical ventilation combined with selective PEEP consistently increases DO_2_, however it requires a surgical procedure (tracheostomy) and multiple ventilators [37].

The lowest individual PaO_2 _measurements in our study ranged from 6.32 to 13.55 kPa (48 to 103 mm Hg) in the 0.5 FiO_2 _group and from 9.21 to 42.1 kPa (70 to 320 mm Hg) in the >0.95 FiO_2 _group. All horses in 0.5 FiO_2 _group had PaO_2 _values below 13.16 kPa (100 mm Hg) at some time during the anesthetic period and 2 of 5 horses had PaO_2 _values below 7.89 kPa (60 mm Hg). Only one of 5 horses in the >0.95 FiO_2 _group had a PaO_2 _below 13.16 kPa (100 mm Hg). All horses in both trials had PaO_2 _values below 7.89 kPa (60 mm Hg) ten minutes after discontinuation of anesthesia but CaO_2 _did not change from preanesthetic levels and was not different between groups at any time suggesting there was no apparent advantage with either technique. Further, recent studies in laterally recumbent isoflurane anesthetized horses have determined that breathing >0.95 FiO_2 _for 90 min does not affect erythrocyte membrane dynamics or structure, blood viscosity or muscular perfusion and that breathing room air (0.21 FiO_2_) decreases skeletal muscle oxygenation [39].

The results of our study, although limited by a relatively small sample size, are similar and consistent with those of others [23,26]. One of our horses developed atrial fibrillation, which could have influenced the development of V/Q and our results. The range of HR and Q for the horse that developed atrial fibrillation was similar in both trials so we do not believe that it affected our results since the development of atrial fibrillation in the absence of cardiac disease, is not necessarily associated with the deterioration of hemodynamics [40]. We did not have the capability of performing the multiple inert gas elimination technique which would have allowed potential differentiation between pulmonary units with low and high V/Q and shunt [23,41,42]. Regardless, the ability to more precisely identify V/Q matching and shunt would not have changes our results. Decreases in atelectasis, Qs/Qt, and P_(A-a)_O_2 _are interesting but unless they result in improvements in PaO_2_, CaO_2_, and DO_2_, their impact is suspect. The risks of inhaling >0.95 FiO_2 _are well documented in other species [43-45] but we could find no reports of pathology associated with the use of >0.95 FiO_2 _in horses anesthetized for clinically relevant durations. To the contrary, isoflurane anesthetized horses breathing >0.95% O_2 _for 90 min demonstrated minimal damage from reactive oxygen species and no alterations in muscular perfusion [39]. The use of submaximal FiO_2 _requires additional equipment and more stringent monitoring thus increasing expense. In the absence of evidence of benefit, the authors do not recommend the use of submaximal 0.5 FiO_2 _in anesthetized horses.

## Conclusion

The purpose of breathing increased FiO_2 _during equine anesthesia is to partially offset the loss of the adaptive mechanisms that are initiated by hypoxemia in awake horses such as increases in ventilation, Q and contraction of the spleen [3]. These mechanisms are obtunded or eliminated during inhalant anesthesia in horses. Our study suggests that decreasing FiO_2 _to 0.5% from maximal levels does not improve pulmonary gas exchange or DO_2 _during inhalant anesthesia in horses. Further studies investigating the benefits of alternative gas mixtures (ex. helium- O_2_) and modes of ventilation in conjunction with changes in FiO_2 _are warranted.

## Methods

### Horses

Five mature horses (one Thoroughbred, one Quarterhorse, three Standardbreds; two geldings, three mares) with a mean body weight of 614 kg bwt (range 578 to 638 kg bwt) were studied. All horses were maintained on pasture and were judged to be overweight based on subjective evaluations of body conditions and round-bellied appearances [33]. The experimental protocols and procedures were approved by the Institutional Animal Care and Use Committee of The Ohio State University and all horses were treated in compliance with NIH Guidelines for the Care and Use of Laboratory Animals.

### Study Design

The study was conducted as a random-ordered 2-way cross-over. A complete blood count (CBC) and serum biochemistry analysis were performed before and 24 hr after each trial. Food, but not water, was withheld for approximately 12 hr before each trial. All horses underwent two separate 3-hour anesthetic episodes separated by at least 10 days. Each horse was randomly assigned to breathe either 100% O_2 _to attain >0.95 FiO_2 _(Group 1) or O_2 _blended with medical grade air to attain 0.5 FiO_2 _(Group 2).

Each horse was confined in a stockade and the hair over the left and right jugular veins was clipped. The skin was surgically prepared for aseptic placement of intravascular and intracardiac catheters. Lidocaine (2%, 1 ml/site) was injected subcutaneously at two sites over the right jugular vein and one site over the left jugular vein. A 14-gauge Teflon catheter was percutaneously placed in the left jugular vein for administration of anesthetic drugs and isotonic electrolyte solutions (10 mL/kg bwt/hr). Two 8F catheter introducers (Catheter introducers CL-07811, Arrow International Inc.) were percutaneously placed in the right jugular vein to facilitate the placement of two catheters: 1) A 7-French specialized thermistor and balloon tipped quadruple lumen catheter (Thermodilution balloon catheter AI-07067, Arrow International Inc.) was positioned so that its distal tip was in the pulmonary artery; and 2) a 110 cm polyethylene (PE) 240 catheter (Intramedic PE-240 tubing, Becton Dickinson and Company) was positioned so its distal tip was in the right atrium. A 20-gauge 1.25 inch catheter (Surflo catheter, Terumo Medical Corporation) was percutaneously positioned in either transverse facial artery or a 19 gauge through the needle catheter (Intracath, Parke, Davis & Company) was inserted into a subcutaneously relocated carotid artery. These procedures facilitated the determination of systolic arterial blood pressure (SABP), diastolic arterial blood pressure (DABP), mean arterial blood pressure (MABP), and collection of samples of heparinized blood samples for the determination of arterial pH (pHa) and blood gas analysis (PaO_2_; PaCO_2_). Heparinized samples of venous blood were anerobically obtained from the pulmonary arterial catheter for determination of venous pH, venous partial pressure of oxygen (PvO_2_), and venous partial pressure of carbon dioxide (PvCO_2_). Proper positioning of all catheters was confirmed by attaching each catheter to a pressure transducer (TruWave Disposable PressureTransducer, Edwards Lifesciences LLC) and visualizing their characteristic pressure waveforms (Datascope Passport Model EL, Datascope Corp.). The scapulohumeral joint was considered the zero pressure reference point. One mL of iced injectate (5% dextrose^j^) solution/15 kg bwt was rapidly injected via the right atrial catheter (PE 240 catheter) for determination of cardiac output (Q; L/min) by thermodilution (Cardiomax III, Columbus Instruments). The value for Q was at each time point was determined by an average of three Q determinations. A base-apex electrocardiogram was used to determine heart rate (HR) and rhythm. The quadruple lumen thermodilution catheter facilitated the determination of Q and MPAP. The PE 240 catheter was used for the administration of iced injectate for Q determinations and for measurement of MRAP. Respiratory rate (RR) was determined by observing chest excursions in standing horses and movement of the anesthetic machine ventilatory bellows during anesthesia.

Horses were allowed to stand in the stockade undisturbed for 10 min following catheter placement. Then, baseline heart rate (HR; beats/min), respiratory rate (RR, breaths/min), Q (L/min), CI (L/kg bwt/min), SABP (mmHg), MABP (mmHg), DABP (mmHg), MPAP (mmHg), and MRAP (mmHg) were measured. Baseline arterial and venous blood samples were obtained anaerobically for determination of hemoglobin levels, oxygen saturation levels, pH and blood gas analysis (PaO_2_; PaCO_2_; PvO_2_; PvCO_2_), and lactate determination (ABL 725 Radiometer America). Packed cell volumes were determined by centrifugation and total protein levels were determined by refractometry (Clinical refractometer J-351, Jorgensen Laboratories Inc). Horses were moved to a padded induction stall and xylazine (1.0 mg/kg bwt, IV) was administered for sedation 5 min after sample collection. Ketamine (2.2 mg/kg bwt) and diazepam (0.1 mg/kg) bwt) were administered as an IV bolus five min after xylazine administration. Once recumbent, horses were positioned in lateral recumbency and a 26 mm diameter ID orotracheal tube was placed in the trachea. Horses were positioned in dorsal recumbency and appropriately padded. The endotracheal tube was connected to a circle anesthetic machine (Model 2800, Mallard Medical, Inc.) primed with the fresh gas mixture to be tested and 3% isoflurane (ISO). An initial total fresh gas flow rate of 9 L/min was delivered for 15 min and was then reduced to a total of 9 mL/kg bwt/min for the remainder of the anesthetic period. Anesthesia was maintained at an end-tidal ISO concentration of 2%. Controlled ventilation (initial tidal volume 15 mL/kg bwt, initial respiratory rate 6 breaths/min) was immediately instituted and adjusted to maintain PaCO_2 _between 40 and 45 mmHg. Inspired and expired concentrations of O_2_, CO_2_, and ISO were determined using a side-stream gas analyzer (Poet IQ2 8500Q, Criticare Systems, Inc.). Dobutamine (1 - 5 μg/kg bwt/min) was administered as needed to maintain MABP between 70 and 90 mm Hg.

Values for HR, SABP, MABP, DABP, MPAP, MRAP, Q, peak inspiratory pressure (PIP; cmH_2_O), inspired and expired O_2 _concentrations (%), inspired and peak expired CO_2 _concentrations (mmHg), end-tidal ISO concentration (%) and heparinized samples of arterial and venous blood were anaerobically obtained for determination of pH, blood gases, hemoglobin, and SO_2 _at 15, 30, 45, 60, 90, 120, 150, and 180 min after induction to anesthesia. Tidal volume (V; Liters) was measured using a digital respirometer (Respirometer Model 00-295, Anesthesia Associates, Inc.) placed between the endotracheal tube and the breathing circuit. Derived variables calculated from measured data included: systemic vascular resistance (dynes sec cm^-5^), cardiac index (CI; L/kg bwt/min) [46], shunt fraction (Qs/Qt; %), arterial and venous oxygen content (CaO_2 _and CvO_2_, respectively; mL/dL), oxygen consumption (VO_2_; mL/kg bwt/min) [46], oxygen delivery (DO_2_; mL/kg bwt/min) [46], arterial-mixed venous oxygen content difference (C_(a-v)_O_2_; mL/dL), oxygen extraction ratio (O_2_ER; %), alveolar dead space (V_d_/V_t_; %), alveolar arterial oxygen tension (P_A_O_2_; kPa), alveolar arterial oxygen tension difference (P_(A-a)_O_2_; kPa), and arterial partial pressure of oxygen/inspired partial pressure of oxygen ratio (PaO_2_-IO_2 _ratio). Values were calculated according to the following equations:

Horses were transported to a padded recovery stall and positioned in left lateral recumbency after the 180 min collection interval. One breath/min was delivered using a demand valve (Equine Demand Valve Model 5040, JD Medical Distributing) until spontaneous ventilation resumed. Once spontaneous ventilation resumed, xylazine (0.2 mg/kg bwt, IV) was administered to provide sedation during recovery. All data was collected 10 min after the anesthetic machine was disconnected and 30 min after the horse attained a standing position. The times from disconnection from the anesthetic machine to the resumption of spontaneous ventilation, first movement, extubation, first attempt to attain a sternal position, sternal recumbency, first attempt to stand, time to standing, and number of attempts to stand were recorded. The quality of recovery was assessed by at least two independent observers using a 10 point scale: *1. Stands on first attempt with minimal effort, minimal ataxia; 2. Stands on first attempt with minimal to moderate effort, mild ataxia; 3. Stands on first or second attempt with great effort and moderate ataxia. Marked weight shifting once standing; 4. 2-3 attempts to stand, moderate effort, slight weight shifting; 5. 2-3 standing attempts, marked instability once standing; 6. Several weak attempts, marked instability once standing; 7. Several weak attempts, resumes recumbency, minor shifting of weight once standing; 8. Several weak attempts, falls easily or resumes recumbency, minor injury to horse; 9. Several violent attempts, falls or resumes recumbency, minor injury to horse; 10. Several violent attempts, resumes recumbency, major injury to horse or personnel*.

### Statistical Analysis

All numerical continuous data are presented as mean ± standard error of the mean. A 2-way ANOVA with repeated measures was used to analyse for treatment effects and interaction. Tukey-Kramer post-test was performed to identify time and within and between treatment differences. Normally distributed recovery data was analyzed using a 1-tailed paired t-test. Recovery score and number of attempts to stand were analyzed using a Wilcoxon signed rank test. A *P *< 0.05 was considered significant.

## Authors' contributions

JAEH participated in the design of the study, data collection, statistical analyses, and manuscript preparation. TKA participated in data collection, statistical analyses, and manuscript preparation. RMB participated in the design of the study, data collection, and manuscript preparation. PL participated in data collection and manuscript preparation. WWM participated in the design of the study and manuscript preparation. All authors have read and approved the final manuscript.
